# Antimicrobial Activity and Chemical Constituents of *Siparuna guianensis* (Siparunaceae)

**DOI:** 10.1002/cbdv.202501388

**Published:** 2025-07-12

**Authors:** Olência Mento Manuensa Vilanculo, Mayra Suelen da Silva Pinheiro, Luana Cardoso de Oliveira, Dayenne Alexa Araújo de Souza, Sandra Valeria Dias Cardoso, Sebastião da Cruz Silva, Patrícia Santana Barbosa Marinho, Andrey Moacir do Rosario Marinho

**Affiliations:** ^1^ Programa De Pós‐graduação em Química Universidade Federal do Sul e Sudeste Do Pará Maraba Brazil; ^2^ Programa De Pós‐graduação em Química Universidade Estadual de Campinas Campinas Brazil; ^3^ Programa De Pós‐graduação em Química Universidade Federal do Pará Belem Brazil

**Keywords:** Aporphine alkaloids, amazon, antimicrobial activity, flavonoids, *Siparuna guianensis*

## Abstract

*Siparuna guianensis* (Siparunaceae) is an Amazonian plant known by the popular name of Negramina. There are several reports of the use of this plant in folk medicine. In the present work, we decided to study its extracts, aiming at the isolation of compounds and verifying their antimicrobial activities. After obtaining the extracts, the compounds were isolated through chromatographic methods and identified by nuclear magnetic resonance and mass spectrometry. The antimicrobial activities were performed through the microdilution method in 96‐hole plates. Six compounds were isolated, two of which belong to the flavonoid class, kaempferol‐3,7‐dimethyl ether (**1**) and herbacetin 3,7‐O‐dimethyl ether (**2**), and four belong to the aporphine alkaloid class: isobulbocapnine (**3**), norlirioferine (**4**), liriodenine (**5**), and anonaine (**6**). Of the six compounds isolated from *S. guianensis*, compounds **3**, **4**, **5**, and **6** are being reported for the first time in the species. The aporphine alkaloid compounds, isobulbocapnine (**3**) and norlirioferine (**4**) are being reported for the first time for the genus *Siparuna*. Compound **6** showed good activity against strains of phytopathogenic bacteria.

## Graphical abstract

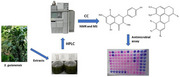

## 1 Introduction

Secondary metabolites, commonly referred to as “natural products” (NPs) (the end products of gene expression), are an essential and respectable source of successful drug leads that originate from the biodiverse floras and faunas of the earth. However, more than 95% of the world's biodiversity has yet to be assessed [1].

Secondary metabolites are also referred to as specialized metabolites or special products [2, 3, 4, 5]. Highlighting phenolic compounds (flavonoids), terpenes, and nitrogenous compounds (alkaloids), they are often lineage‐specific and help plants interact with the biotic and abiotic environment [2, 4, 6].

These natural products, as they are factors of interaction between microorganisms, often present interesting biological activities. Many are of commercial importance in the food, agronomic, and perfumery sectors, among others. From a pharmaceutical point of view, the greatest interest derives mainly from the large number of pharmacologically important substances [7].

Plants have developed a vast and rich chemistry to sustain their sessile lifestyles. Man has exploited this natural resource since Neolithic times, and today, chemical products derived from plants are exploited for a multitude of applications. Among the plants, we can highlight *Siparuna guianensis*.

*S. guianensis* Aublet is a plant that belongs to the Siparunaceae family. It was the first species of Siparun described and illustrated by Aublet in Histoire des plantes de La Guiana Françoise (1775). It occurs from Nicaragua, throughout northern South America to Paraguay, in plains of elevated primary and secondary forests, with heights of 1.20 m, rarely 1.40 m [8]. It is also known as capitiú and negramina, and is present in almost all of Brazil, but more frequently in northern Brazil.

Chemical and ethnobiological studies on *S. guianensis* are limited, with only a few reports of traditional medicine in Central and South America, including the use of leaf juice against fevers and as a postpartum antibiotic. In Brazil, *S. guianensis* is used in folk medicine, and its leaves and flowers are considered carminative, aromatic, antidyspeptic, and diuretic, in addition to having stimulating and febrifuge properties [9]. It is an anti‐inflammatory, stimulant, and relieves rheumatism [10]. It is also used to relieve sinusitis. Its dried leaves, ground and mixed with fire, produce smoke which, when smelled, cures malina [11].

In this research, the secondary metabolism of *S. guianensis* (Negramina) from the Amazon Region was studied, aiming at the prospecting and isolation of bioactive chemical substances as well as evaluating their antimicrobial activity.

## 2 Results and Discussion

### 2.1 High‐Performance Liquid Chromatography with Diode Array Detection Analysis of Extracts

**FIGURE 1:**
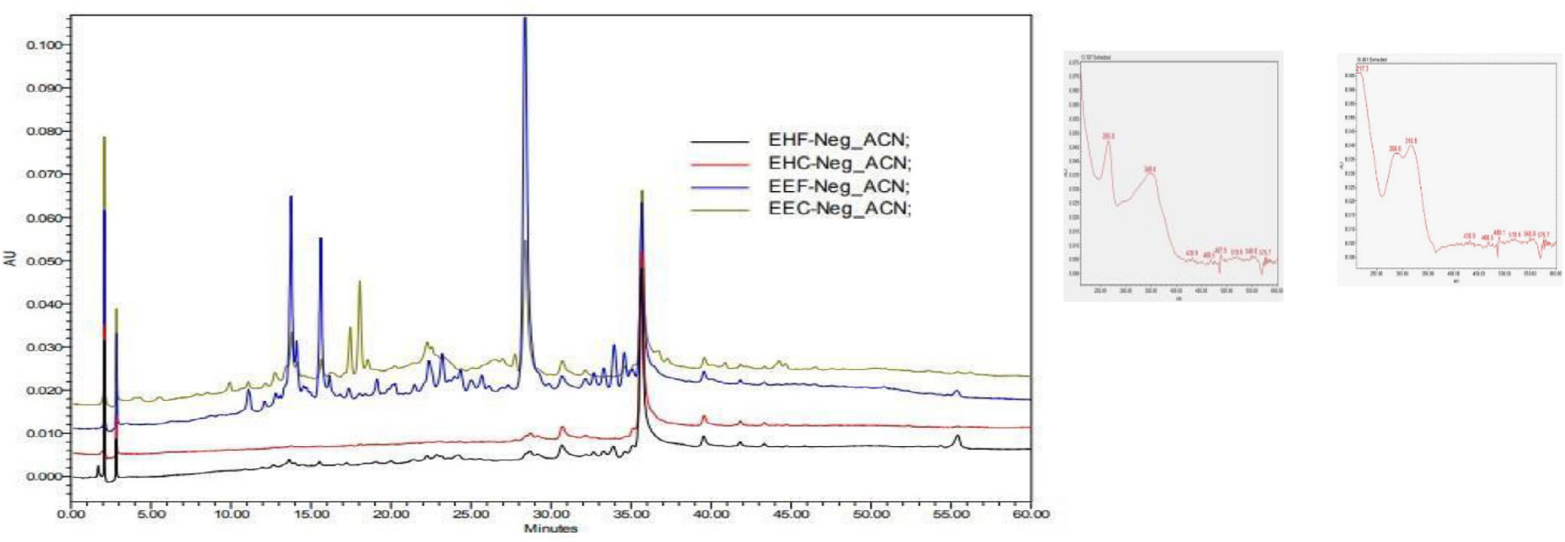
Chromatograms obtained by high‐performance liquid chromatography with diode array detection (HPLC‐DAD) of the crude extracts: (hexanic leaf extract [EHF], hexanic stem extract [EHC], ethanolic leaf extract [EEF], and ethanolic extract of the stem [EEC]), λ = 265 nm.

The crude extracts of the plant parts (hexanic leaf extract [EHF], hexanic stem extract [EHC], ethanolic leaf extract [EEF], and ethanolic extract of the stem [EEC]) were subjected to high‐performance liquid chromatography with diode array detection (HPLC‐DAD) analysis in which, of the quantitative results, only the chromatograms of the EEF and EEC extracts showed the presence of secondary metabolites and, through the ultraviolet (UV) spectrum, a suggestion of which class these metabolites belong to (Figure 1).

UV absorption spectra (absorption peaks at λ of 265 and 350 nm) are characteristic of phenolic compounds, more specifically flavonols [12]. Peak with bands at *λ*_max_ 217.3, 286, and 319 nm, suggests the presence of aporphine alkaloids. The UV spectrum showed three absorption bands at *λ* = 220, 283, and 304 nm [13].

### 2.2 Identification of Isolated Compounds

**FIGURE 2:**
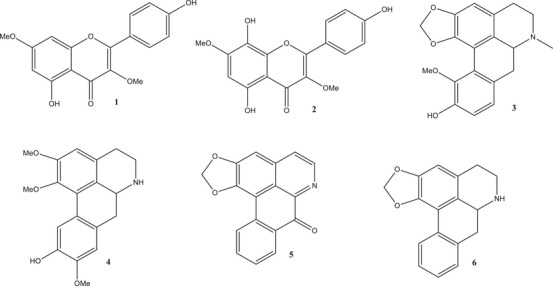
Compounds isolated from *S. guianensis*.

After HPLC analysis of the negramina extracts, it was decided to isolate the compounds from the EEC, as it presented a better chromatographic profile. Thus, the EEC extract was successively fractionated by CC (silica), leading to the isolation of the compounds (Figure 2). The compounds were identified through analysis of their NMR and MS spectra and by comparison with literature data. As observed by HPLC‐DAD analysis, which indicated the presence of flavonoids and aporphine alkaloids, two substances from the flavonoid class, kaempferol‐3,7‐dimethyl ether (**1**) and herbacetin 3,7‐O‐dimethyl ether (**2**), and four from the aporphine alkaloid class: isobulbocapnine (**3**), norlirioferine (**4**), liriodenine (**5**), and anonaine (**6**) were isolated [14, 15, 16, 17, 18].

Of the six compounds isolated from *S. guianensis*, compounds **3**, **4**, **5**, and **6** are being reported for the first time in the species. The aporphine alkaloid compounds Isobulbocapnine (**3**) and Norlirioferine (**4**) are being reported for the first time in the genus Siparuna.

### 2.3 Antimicrobial Assays

Both the extracts and the isolated compounds were tested against two different types of bacteria. The first set refers to bacteria pathogenic to animals and humans (Table 1), which are: *Escherichia coli* (ATCC 25922), *Bacillus subtilis* (ATCC 6633), *Pseudomonas aeruginosa* (ATCC 27853), *Salmonella typhimurium* (ATCC 140228), and *Staphylococcus aureus* (ATCC 25923). The second set refers to phytopathogenic bacteria, that is, those that cause diseases in plants (Table 2), and these strains of *Xanthomonas axonopodis* pv. passiflorae are responsible for causing diseases in passion fruit plants [19]. The results are shown in Tables 1 and 2.

**TABLE 1:** Results of the antimicrobial test with pathogenic bacteria of the extracts and substances.

Samples tested	Pathogenic bacteria			
	Bs	Sa	Pa	St
**EHF**	—	—	—	—
**EHC**	—	—	—	—
**EEF**	—	—	—	—
**EEC**	—	—	—	—
**1**	250	125	—	125
**3**	—	—	—	—
**4**	125	—	—	—
**5**	125	—	—	—
**6**	31.25	62.5	—	—
**A**	3.91	3.91	3.91	3.91
**T**	3.91	3.91	3.91	3.91
**C**	3.91	3.91	3.91	3.91

*Note*: Values are in µg/mL.

A—Amoxicillin; T—Tetracycline; C—Cefadroxil; B.s—*Bacillus subtilis*; S.a—*Staphylococcus aureus*; P.a—*Pseudomonas aeruginosa*; S.t—*Salmonella typhimurium*.

**TABLE 2:** Results of antimicrobial assay with pathogenic bacteria of extracts and substances.

Samples tested	Phytopathogenic bacteria			
	Xap 1	Xap 2	Xap 3	Xap 4
**EHF**	—	—	—	—
**EHC**	250	—	—	125
**EEF**	250	250	250	—
**EEC**	—	—	—	—
**1**	250	250	250	250
**2**	—	—	—	—
**3**	—	—	—	—
**4**	31.25	250	250	250
**5**	31.25	250	250	250
**6**	31.25	125	62.5	31.25

*Note*: Values are in µg/mL. Xap—*X. axonopodis* pv. Passiflorae. **1**, **2**, **3**, and **4** are different strains.

The results obtained in the tests showed that both the extracts and the compounds presented selectivity for phytopathogenic bacteria, where alkaloid **6** was the substance with the best activity, inhibiting bacterial growth up to a concentration of 31.25 µg/mL. The activity observed for **6** is of great importance since it suggests selectivity for phytopathogenic bacteria, which opens the possibility of developing an agricultural product capable of controlling the growth of Xap and thus reducing the damage caused by this bacteria to macaruja plantations [20] and at the same time not interfering with the development of non‐phytopathogenic bacteria.

## 3 Conclusions

The study of *S. guianensis* led to the isolation of compounds from the classes of flavonoids and aporphine alkaloids, which are commonly found in the genus. However, alkaloids **3**, **4**, **5**, and **6** are being reported for the first time in the species. The aporphine alkaloid compounds isobulbocapnine (**3**) and norlirioferine (**4**) are being reported for the first time in the genus Siparuna. The compound anonaine (**6**) showed good activity against X. axonopodis pv. Passiflorae.

## 4 Experimental

### 4.1 Plant

Leaves and stems of negramina were collected in December 2023 at the Marabá Zoobotanical Park, located in the state of Pará, city of Marabá, highway BR 155. An exsiccate is deposited in the Herbarium South and Southeast of Pará with code Sepa 0030

### 4.2 Obtaining Extracts

The collected fresh material (leaves and stems) was dried in an oven with air circulation at 40°C. After drying, the material was ground in a knife mill until powder was obtained, resulting in 2.0 kg of powder for each part of the plant. For the extraction process, 100 g of powder from each part of the plant was macerated in 500 mL of hexane for 72 h. Then, the solvent was filtered using a Büchner funnel, concentrated in a rotary evaporator, and subjected to complete evaporation of the solvent to obtain the hexane extract. This procedure was performed in triplicate. After extraction with hexane, the same material was reused for a new extraction process, following the same steps, but using ethanol as the solvent, resulting in the ethanolic extract.

### 4.3 HPLC Analysis of Extracts

For the analysis, the crude extracts were prepared at a concentration of 1 mg/mL, being solubilized in HPLC‐grade methanol, sonicated in an ultrasound bath for 10 min, and subsequently transferred to 1.5 mL vials. A Waters e2695 Alliance Liquid Chromatograph (Waters) coupled to a Waters 2998 Diode Array Detector (Waters) was used. The system used in the HPLC‐DAD analysis was a Sunfire octadecyl silane analytical column and gradient elution with water/acetonitrile (95:5→0:100) for 60 min, with a flow rate of 1.0 mL/min and an injection volume of 20 µL. A scan was performed in the range of 210–600 nm with monitoring of the wavelengths of 220, 240, 265, and 348 nm. The column oven temperature was maintained at 40°C. Chromatograms were generated using the Empower program (Waters).

### 4.4 Fractionation of Extracts and Isolation of Compounds

EHC, EHF, EEF, and EEC were analyzed by analytical thin layer chromatography (ATC) using 200 × 200 mm MF254 silica gel coated aluminum plates (Agela Technologies) as the stationary phase and systems with hexane (Hex) and ethyl acetate as the mobile phase. The plates were developed in a UV light chamber (Boitton Instrumentos) at wavelengths of 254 and 365 nm, and in an acidic solution of ceric sulfate. Isolated compounds were identified by NMR and MS data.

### 4.5 Antimicrobial Assays

The strains of the pathogens *B. subtilis* (ATCC 6633), *P. aeruginosa* (ATCC 27853), *S. typhimurium* (ATCC 140228), and *S. aureus* (ATCC 25923) were provided by the Evandro Chagas Institute (IEC). The strains of the phytopathogen *Xanthomonas axonopodis* pv. passiflorae were provided by the Brazilian Agricultural Research Corporation of Pará (Embrapa Amazônia Oriental): *X. axonopodis* pv. passiflorae (Xap 1), *X. axonopodis* pv. passiflorae (Xap 2), *X. axonopodis* pv. passiflorae (Xap 3), *X. axonopodis* pv. Passiflorae (Xap 4). In the 96‐well ELISA plates, 100 µL of MH broth for pathogenic bacteria and MB1 for phytopathogenic bacteria were added to each well. Then, 100 µL of the solution containing the samples to be tested was added to the first well (well A), and this solution was homogenized. After this, successive dilutions were performed, removing 100 µL from the first well (well A) and transferring this volume to the next well (well B), homogenizing. This procedure was repeated until the penultimate well of the plate, from which 100 µL of solution was removed and discarded. In each well, 5 µL of the bacterial suspension was added. The last line of the plate represents the negative control that contains only the MB1/BHI broth and the bacterial suspension. The plates were incubated for 24 h at the temperatures of 37 or 28°C according to the type of pathogen. The results were read by adding 10 µL of 2% TTC solution (2,3,5‐triphenyltetrazolium chloride) prepared with sterile distilled water.

## Author Contributions

**Olência Mento Manuensa Vilanculo**: collected the botanical material, obtained and isolated the compounds, wrote the manuscript. **Mayra Suelen da Silva Pinheiro**: collected the botanical material, obtained and isolated the compounds, performed MS analysis. **Sebastião da Cruz Silva**: collected the botanical material, obtained and isolated the compounds. **Dayenne Alexa Araújo de Souza, Sandra Valeria Dias Cardoso and Luana Cardoso de Oliveira**: performed HPLC analysis and performed antimicrobial assays. **Patrícia Santana Barbosa Marinho**: performed HPLC and NMR analysis. **Andrey Moacir do Rosario Marinho**: performed antimicrobial assays, wrote the manuscript, and supervised the work.

## Conflicts of Interest

The authors declare no conflicts of interest.

## Data Availability

Data available on request from the authors.
